# Bioemulsifiers Derived from Microorganisms: Applications in the Drug and Food Industry

**DOI:** 10.15171/apb.2018.023

**Published:** 2018-06-19

**Authors:** Mahmood Alizadeh-Sani, Hamed Hamishehkar, Arezou Khezerlou, Maryam Azizi-Lalabadi, Yaghob Azadi, Elyas Nattagh-Eshtivani, Mehdi Fasihi, Abed Ghavami, Aydin Aynehchi, Ali Ehsani

**Affiliations:** ^1^Student Research Committee, Department of Food Sciences and Technology, Faculty of Nutrition and Food Sciences, Tabriz University of Medical Sciences, Tabriz, Iran.; ^2^Drug Applied Research Center, Tabriz University of Medical Sciences, Tabriz, Iran.; ^3^Student Research Committee, Tabriz University of Medical Sciences, Tabriz, Iran.; ^4^Student Research Committee, Faculty of Nutrition and Food Sciences, Tabriz University of Medical Sciences, Tabriz, Iran.; ^5^Department of Food Sciences and Technology, Faculty of Nutrition and Food Sciences, Tabriz University of Medical Sciences, Tabriz, Iran.

**Keywords:** Bioemulsifiers, Emulsion stability, Biosurfactants, Microorganism

## Abstract

Emulsifiers are a large category of compounds considered as surface active agents or surfactants. An emulsifier acts by reducing the speed of chemical reactions, and enhancing its stability. Bioemulsifiers are known as surface active biomolecule materials, due to their unique features over chemical surfactants, such as non-toxicity, biodegradability, foaming, biocompatibility, efficiency at low concentrations, high selectivity in different pH, temperatures and salinities. Emulsifiers are found in various natural resources and are synthesized by Bacteria, Fungi and Yeast. Bioemulsifier’s molecular weight is higher than that of biosurfactants. Emulsion’s function is closely related to their chemical structure. Therefore, the aim of this paper was to study the various bioemulsifiers derived from microorganisms used in the drug and food industry. In this manuscript, we studied organisms with biosurfactant producing abilities. These inexpensive substrates could be used in environmental remediation and in the petroleum industry.

## Introduction

As it is clear, oil and water are incompatible. The mixture of oil and water lead to the production of an emulsion. When an emulsion stays still for a while, oil droplets begin to separate from water. In this regards, emulsifiers are used to stop this process. In fact, emulsifiers are used to prevent the emulsion from breaking. Examples of emulsions currently used in the food industry include milk, butter, margarine, mayonnaise and ice cream. Emulsifiers are a large category of compounds also known as surface active agents or surfactants. The word surfactant is used for molecules that migrate to the surface between phases.^1-6^

Bioemulsifiers are higher in molecular weight compared to biosurfactants since they are complex mixtures of heteropolysaccharides, lipopolysaccharides, lipoproteins and proteins.^7^ Emulsifiers have double lipophilic and hydrophilic properties. On the other hand, emulsions are either oil-in-water (O/W) or water-in-oil (W/O). In oil emulsions, small droplets of oil form the dispersed phase and discrete in water, while in water emulsions, they are distrebuted as small droplets of water in oil.^8,9^ Adding an emulsifier to an unmixable compound, reduces surface tension between the two phases and prevents it from separating. Therefore, the two liquids are able to form an emulsion. Since an emulsion consists of water-soluble and oil-soluble fragments, an emulsifier is placed on the surface of the area where the two liquids (water and oil) are connected. The water-soluble fragment ambulates towards the water fragment and the fat-soluble fragment places near the oil.^4,6,9-12^

Emulsifiers are substances that increase the uniformity of nutrients, such as fatty acids, fat-soluble vitamins, and amino acids. The function of emulsions is closely related to its chemical structure.^9^ Physiologically, in animal’s digestive system, the presence of bile salts, benefits fat absorption. Emulsifiers are surfactant materials widely used in food products.^9,13^ Hydrophilic characteristics (water-friendliness) and lipophilic (lipid-friendliness) emulsifiers are sometimes referred as hydrophilic/lipophilic equilibrium (HLB), indicating the rate of emulsifier’s inclination towards water or oil.^14-16^ Emulsifiers are embedded in fat droplets and prevent the protein layer from collapsing. Other functions of emulsifiers in the food industry include:

1) Starch reaction: most emulsifiers have a lean fatty acid layer in their molecule which form an amylose mixture. This feature is very important in delaying bread and bakery products staling and reducing their adhesion to staple products such as potato puree and pasta.^10,11,17,18^

2) Generating interactions with proteins: emulsifiers have ionic structure which react with proteins in food products and produce a modifiable structure. For example, they respond to the gluten present in wheat and increase protein elasticity, thereby increasing bakery products volume.^10,14,19^

3) Adhesion correction: some emulsifiers are added to food products containing sugar crystals that are scattered in fat and, by coating on glucose crystals, reduce adhesion. This feature affects the fluidity of molten chips and prevent fat appearance on the surface of chocolate.^11,20-22^

4) Creating foam: emulsifiers with saturated fatty acids stabilize the bottommost surface of aqueous solutions. Therefore, it is an important factor in creating foam in raw instant desserts.^4,11,21^

5) Tissue modification is a complex process that is performed on starch and reduces breakdown. For example, homogeneity usually occurs in pasta, bread and bakery products.^2,11^

6) Modifying the dispersion of liquids in another liquid in order to formulate clear solutions: many greed and colors require emulsifiers for solving.^11,14^

**Figure 1:**
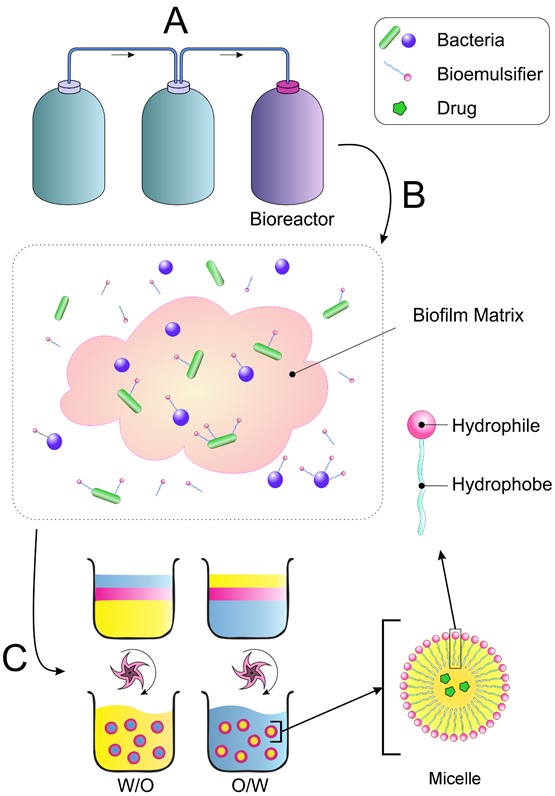
The schematic and mechanism of action of bioemulsifiers in emulsion systems. A: The cultivation, preparation, purification and isolation of microorganisms producing bioemulsifiers. B: Bioemulsifiers produced by microorganisms. C: Emulsion production, adding bioemulsifiers to emulsion systems and assessing their stability.

Today, due to emulsifier effect on human health and limited resources as well as expensivity, researchers have produced emulsifiers using natural resources, especially microorganisms.^19^ According to findings, many microorganisms are able to produce compounds with emulsifying properties.^10^ A number of these bioemulsifiers have been licensed by the International Organization for Animal Health, including WHO; but most of these compounds have been studied from a nutritional point of view. A large number of biomolecules are also used in the oil, food, drug and chemical industries.^11,17^ The schematic and mechanism of action of important emulsifiers produced by microorganisms using biotechnological processes are presented in Figure 1.

### Bioemulsifiers derived from yeast and fungi

#### Mannoprotein

Mannoprotein bioemulsifier is a glycoprotein with a molecular weight of about 14,000 to 15,800 Dalton. Within the cellular wall of *Saccharomyces spp.* and *Kluyveromyces marxianus* of yeast, mannoprotein molecules are present in glucan, networks, and released from the cell wall of yeast using pressurized heat treatments. This bioemulsifier is able to stabilize oil-in-water emulsions (O/W). According to researchers, bioemulsifiers can be used for producing mayonnaise along with carboxymethyl cellulose (CMC), instead of using expensive ingredients such as ginseng for mayonnaise formulation. Bakery's yeast (*Saccharomyces cerevisiae*) is an affordable, inexpensive and non-toxic source used for producing this bioemulsifier. Mannoprotein is stable in pH = 3-11. Removing mannoprotein molecules from bakery yeast cell is possible using thermal and enzymatic processes (β*-*1 and 3 glucanases). Mannoprotein molecules are formed from a polypeptide chain with short and long mannose links. When the protein portion of the mannoprotein molecule is detached by the protease enzyme, the mannoprotein emulsifier disappears. In an industrial scale, this bioemulsifier is active at concentrations equal to or greater than 5% sodium chloride.^10-12,19,23,24^

### Liposan

Liposan bioemulsifier is a water-soluble emulsifier obtained from extracting organic solvents fermented by *Candida lipolytica* yeast. Liposan is produced in the extracellular layer and consists of 83% carbohydrate and 17% protein. The presence of protein fractions in the bioemulsifier polymer molecule is essential for its emulsifying properties. The carbohydrate part is a heteropolysaccharide, composed of glucose, galactose, galactose-amine and galacturonic acid molecules. The maximum liposomal properties of liposan are observed at pH = 2-5. Liposan is resistant to temperatures less than 70 °C, but after heating at 100 °C for one hour, 60% of its emulsifiable strength reduces. Liposan causes the stability of various types of emulsions in oil, such as hydrocarbons, vegetable oils including cottonseed, soybean, sunflower, corn, ground, safflower and olive oil.^10,25-30^

### *Candida tropicalis* yeast

During the fed-batch process, *Candida* yeast species produce an extracellular bioemulsifier. This bioemulsifier is very effective in fixing emulsions of many types of hydrocarbons, especially aromatic compounds. The amount of emulsifier produced and its activity increases during fermentation by limiting nitrogen (N) source. Extracting this bioemulsifier from *Candida tropicalis* cells using hot water shows better results in terms of increasing emulsion strength.^11,14,17,31-34^

### *Rhodotorula* yeast

This bioemulsifier is an extracellular emulsifier produced by the *Rhodotorula glutinis* yeast. It is formulated during fed-batch fermentation and glucose utilization under limited nitrogen conditions at 30 °C and pH = 4.^32,33,35-37^

### *Phaffia* yeast

*Phaffia rhodozyma* is a *basidiomycetous* pink yeast. It has been known as a natural source of astaxanthin and many other nutrients. Also, it is currently being used as an ingredient in feeds. It grows on carbohydrate, hydrocarbon and a mixture of carbohydrate and lipid polymers. Fermentation is accomplished in a culture medium chain with sucrose as the carbon source for 3 days at 22 °C and centrifuged at 150 rpm. Experiments have shown that adding sodium citrate, stimulates bioemulsifiers production.^10,11,38,39^ Table 1 and 2 show bioemulsifiers produced by yeast and fungi.

**Table 1:** List of bioemulsifiers producing yeast

Microorganism’s	Bioemulsifiers	Ref.
*Torulopsis petrophilum*	Sophorolipids	^40^
*Torulopsis apicola*	Sophorolipids	^41^
*Pseudozyma rugulosa*	Mannosylerythritol lipids	^42^
*Pseudozyma aphidis*	Mannosylerythritol lipids	^43^
*Kurtzmanomyces sp.*	Mannosylerythritol lipids	^44^
*Kurtzmanomyces sp. I-11*	Mannosylerythritol lipids	^45^
*Debaryomyces polymorphus*	Carbohydrate protein-lipid complex	^32^
*Saccharomyces cerevisiae*	Mannoprotein	^23^
*Kluyveromyces marxianus*	Mannoprotein	^12^
*Rhodotorula glutinis*	Polymeric bioemulsifier	^46^

**Table 2:** List of bioemulsifiers producing fungi

Microorganism’s	Bioemulsifiers	Ref.
*Candida tropicalis*	Mannan-fatty acid	^47^
*Candida lipolytica Y-917*	Sophorous lipid	^39^
*Candida utilis*	NDA	^39^
*Candida ingens*	Fatty acids	^48^
*Candida lipolytica UCP0988*	Carbohydrate-protein-lipid complex	^49^
*Candida tropicalis*	Liposan	^28^
*Candida bombicola*	Sophorolipids	^50^
*Candida (torulopsis) apicola*	Sophorolipids	^51^
*Candida lipolytica ATCC 8662*	Carbohydrate-protein complex	^52^
*Penicillium chrysogenum*	Polyketide derivative	^53^
*Yarrowia lipolytica IMUFRJ 50682*	Carbohydrate-protein complex	^27,54^
*Yarrowia lipolytica NCIM 3589*	Bioemulsifier	^54^
*Yarrowia lipolytica IMUFRJ 50682*	Yansan	^55^
*Ustilago maydis*	Cellobiose lipids	^56^
*Candida sphaerica UCP0995*	Sophorolipids	^57^
*Candida. glabrata UCP0995*	Sophorolipids	^58^
*Pseudomonas. aeruginosa*	Rhamnolipids	^59^

### Bioemulsifiers derived from bacteria

#### Lauryl fructose

This bioemulsifier is produced by the lipase enzyme obtained from *Pseudomonas spp.*^60^ in a culture media containing dry pyridine. This bioemulsifier has emulsification properties for a variety of hydrocarbons, edible oils, and oil based oils such as margarine and shortening. In a water-containing environment, Laura fructose reduces surface tension from 72 to 29 (MN / m). Also, it reduces the intermolecular reaction of water and hydrocarbons from 50 to 6 when combined with water-insoluble oil compounds.^10,11,61-63^ In Table 3, bio-emulsifiers produced by bacteria are presented.

### Alasan or E-KA53

Alasan is a biomolecular bacterium produced by the *Acinetobacter radioresistant* bacteria. The molecular composition of this complex bioemulsifier consists of polysaccharides and proteins with high molecular weight (100,000 - 230,000 Daltons). If the protein portion is damaged and digested using proteolytic enzymes, the bioemulsifier polymer turns into a thick polysaccharide and loses its emulsifying properties. Heating with alcohol at 50 °C may lead to 2.5 times increase in polysaccharide concentration, while the protein portion and the emulsifying properties of the molecule remain unchanged. Heating at 60-90 °C reduces viscosity and increases emulsifying activity as much as 5.8 times than the initial value. The emulsifying properties of alasan are affected by pH and magnesium ion concentrations. This bioemulsifier is an extracellular product and is used extensively in the food industry. Alasan is produced during *Acinetobacter* bacterium fermentation in a fed-batch fermentation system. The ultimate product consists of 2.2 g of emulsifier per liter of culture fluid. Alcohols stabilize a wide range of oil-in-water (O/W) emulsions, such as n-alkanes, alkaline compounds, liquid paraffin, soybean oil, coconut oil and raw oils. The emulsifying activity of alasan increase approximately 2-3 times, when heated at 100 °C under neutral or alkaline conditions. This bioemulsifier is completely active in pH = 3.3-9.2 and its maximum emulsifier activity is at pH = 5. Magnesium ions increase the activity of emulsifiers both at lower (3.3-4.5) and higher pH (5.5-9.3) than that of the optimal pH. Alasan activity is higher in an environment containing 20 mL citrate than that of same concentrations of acetate or Tris-HCl. According to studies, we can indicate that this bioemulsifier is a high molecular weight anion heteropolysaccharide combined with a protein component such as alanine.^10,11,21,64-70^

**Table 3:** List of bioemulsifiers producing bacteria

**Microorganism’s**	**Bioemulsifiers**	**Ref.**
*Pseudomonas fluorescens*	Viscosin	^71^
*Pseudomonas aeruginosa*	Rhamnolipids	^72^
*Pseudomonas fluorescens*	Carbohydrate-lipid complex	^39^
*Bacillus amyloliquefaciens,*	Surfactin/Iturin	^73^
*Bacillus subtilis*	Subtilisin	^74^
*Bacillus subtilis*	Lichenysin	^75^
*Bacillus licheniformis K51, Bacillus subtilis*	Peptide lipids	^76^
*Bacillus pumilus A1*	Rhamnolipids	^77^
*Bacillus* sp. *AB-2*	Hydrocarbon-lipid-protein	^78^
*Acinetobacter calcoaceticus*	Emulsan	^79^
*Acinetobacter radioresistens*	Alasan	^39^
*Acinetobacter calcoaceticus RAG1*	Emulsan	^80^
*Rhodococcus erythropolis*	Glycoprotein	^81^
*Rhodococcus* sp. *33*	Uronic acids	^82^
*Cyanobacteria*	Trehalose dicorynomycolate	^83^
*Clostridium pasteurianum*	Polysaccharide	^84^
*Debaryomyces polmorphus*	Whole cell	^85^
*Halomonas eurihalina*	Carbohydrate-lipid complex	^86^
*Halomonas*	Emulsifier HE39 & HE67	^87^
*Lactobacillus paracasei*	Glycoprotein	^88^
*Leuconostoc mesenteriods*	Dextran	^89^
*Serratia marcescens, Serrated rubidea*	Serrawettin	^90^
*Bacillus pseudomycoides BS6*	Lipopeptide	^91^
*Pseudomonas. cepacia CCT6659*	Rhamnolipids	^92^
*Bacillus. licheniformis R2*	Lipopeptide	^93^

### Emulsan

Emulsan is an extracellular poly-anionic bioemulsifier produced by *Acinetobacter calcoaceticus RAG 1* bacteria. In fact, emulsan is a lipoheteropolysaccharide polymer containing D-galactose-amine produced during the stationary phase. This bioemulsifier is a poly-anionic and amphiphilic compound which is able to stabilize the hydrocarbon emulsion in water by creating a very thin layer between the hydrocarbon droplets and water. Maximum concentration is obtained when culture media containing 12 carbon-based fatty acids are used as the carbon source. Emulsan production is possible with fermentation methods such as batch, chemo-stat, immobilized cell system and self-cycling fermentation (SCF). Based on SCF methods, bioemulsifier production could increase about 50 times compared to the batch method. Another type of emulsan considered as bio-emulsion is produced by *Acinetobacter calcoaceticus*, which is used in the formulation and production of soft cheese and ice creams as well as creams and skin-protecting materials. Different types of emulsions produced from these bacteria include alpha-amyloemulsan, Apo-alpha-oleo-emulsan, and beta-emulsan, which are used for treating skin infections are widely used in the food industry. These compounds are mostly poly-anionic lipoheteropolysaccharides that are produced by different species of *Acinetobacter venetianus rag-1t* ATCC 31012- and *Streptomyces* NRRL.B- 15615, NRRL.B-15847 or ATCC 31926 and few other species.^7,70,94-98^

### Cyanobacteria

A variety of *cyanobacteria* (Genus phormidium, ATCC 39161) (Oscillatoriales) bacterium produce bioemulsifiers that can be used for producing hydrocarbon and oil emulsions in a fluid environment such as water. This bacterium is obtained from using precise separation methods from riverside water, which subsequently grow on a suitable culture medium under favorable conditions and produce an extracellular bioemulsifier sphincter. The molecular weight of this polymeric bioemulsifier is more than 200,000 Dalton. According to chemical tests, it contains sugars, fatty acids, and a protein fractions. Also, more accurate tests using IR spectrophotometry have shown that it is contains amide, carboxylic and amino groups. This bioemulsifier is used for producing various types of oil-in-water emulsions (O/W).^99-101^

### Pseudomonas cepacia bacteria

This bioemulsifier which in terms of molecular characteristics, is considered as a mixture of glycolic acid, is produced after *Pseudomonas cepacia* bacteria growth and propagation on sunflower oil (as the carbon source) medium. The production of this bioemulsifier is carried out by *Pseudomonas cepacia* bacteria by adding 1.7 % of sunflower oil per liter of culture medium when oxygen and nitrogen levels are controlled. This bioemulsifier is used as a natural source of disintegrating agents used for decomposing and neutralizing polychlorinated biphenyls, especially polychlorinated biphenyls. Polychlorinated biphenyls are toxic and with carcinogenic compounds produced by pesticides during various chemical processes which are able to contaminate industrial wastewater and cultivated soils.^102-105^

### Bacillus stearothermophilus

During growth,* Bacillus stearothermophilus VR-8* produces an extracellular bioemulsifier on a medium containing 4% crude oil. The optimum temperature for producing this bioemulsifier is 50 °C, which at this temperature, 0.6 gr/L bioemulsifier is produced. This emulsifier is purified by acetone and dialysis and contains 46% protein, 16% carbohydrate and 10% fat. Its emulsifier activity is stable over a wide range of temperatures, i.e. 50-80 °C, and pH = 2-8, and salt concentrations (5% chlorine, 5% calcium chloride or 1% chlorine magnesium). The emulsifying properties of this bioemulsifier are related to its stability in a wide range of pH (liposomal at pH = 2-5 and a maximum of 70 °C) and temperatures. This emulsifier is used for removing crude oil from reservoirs and eliminating the remaining oil of crude oil tanks. Hereby, recovery of crude oil, increases by complete scouring of oil remained on storage chamber.^9,106-110^

### Sphingomonas bacteria

Presence of polycyclic aromatic hydrocarbons (PAH) in water resources due to their low solubility, is somewhat problematic. When the molecular weight of these compounds (PAH) are reduced, available microorganisms metabolize them. A number of degrading bacteria have been isolated from multi‏-ring aromatic hydrocarbons of contaminated soils, which produce bioemulsifiers and active compounds, most notably strain No. 107 of *Sphingomonas* bacteria. This bacterium grows on culture media containing a variety of aromatic hydrocarbon compounds and create clear spots on the medium.‏ Also in liquid culture media, this bacterium uses aromatic hydrocarbons as the main source of energy and carbon. Notably, this bioemulsifier has emulsifying properties similar to high molecular weighting polycyclic hydrocarbons.^111-114^

## Discussion

Biosurfactants have received great attention due to their safety and biodegradable properties. Although biosurfactants have various functions,their practical application is limited. Biosurfactants worldwide production was approximately 17 million tons in 2000 and is expected to have a growth rates of 3-4% per year globally. Biosurfactants have many advantages in comparison to synthesized components, such as, biodegradability (easily decomposed by microorganism), low toxicity (Effective Concentration =50), availability of raw materials (produced from cheap materials), physical factors (components which are not affected by temperature, pH and ionic strength tolerances), surface and interface activity (lower surface tension) ,biocompatibility and digestibility, commercial laundry detergents, bio pesticide, medical function (antimicrobial activity, anti-cancer activity, anti-adhesive agents, immunological adjuvants, antiviral activity, gene delivery), food processing industry, cosmetic industry and increasing oil recovery. Considering, biosurfactants applications and their affect on nutrient, micronutrient and environmental factors, their production still remains a challeng. It is expected that in the near future, a new strain of microorganisms will be developed for using as biosurfactants in industries.

## Conclusion

Nowadays, emulsifiers are widely used in the food and drug industry. Therefore, using emulsifiers derived from natural resources are preferred to synthetic emulsifiers because of their nutritional benefits. As a result, using bioemulsifiers derived from microbial sources are beneficial and may be a significant alternative for synthesized emulsifiers. Thus, they can be used efficiently in the food and drug industry in acceptable and recommended quantities.

## Acknowledgments

This review was conducted at Tabriz University of Medical Sciences, Tabriz, Iran.

Compliance with Ethical standards.

## Ethical Issues

This article does not contain any studies with human participants or animals performed by any of the authors.

## Conflict of Interest

The authors declare no conflict of interests.
